# Isolated IgA Anti-**β**2 Glycoprotein I Antibodies in Patients with Clinical Criteria for Antiphospholipid Syndrome

**DOI:** 10.1155/2014/704395

**Published:** 2014-03-23

**Authors:** Raquel Ruiz-García, Manuel Serrano, José Ángel Martínez-Flores, Sergio Mora, Luis Morillas, María Ángeles Martín-Mola, José M. Morales, Estela Paz-Artal, Antonio Serrano

**Affiliations:** ^1^Servicio de Inmunología, Instituto de Investigación Hospital Universitario 12 de Octubre, Avenida Córdoba s/n, 28041 Madrid, Spain; ^2^Servicio de Nefrología, Instituto de Investigación Hospital Universitario 12 de Octubre, Avenida Córdoba s/n, 28041 Madrid, Spain; ^3^Servicio de Reumatología, Instituto de Investigación Hospital Universitario 12 de Octubre, Avenida Córdoba s/n, 28041 Madrid, Spain; ^4^Servicio de Hematología, Instituto de Investigación Hospital Universitario 12 de Octubre, Avenida Córdoba s/n, 28041 Madrid, Spain; ^5^Sección de Inmunología, Universidad San Pablo-CEU, Campus de Monteprincipe, 28668 Madrid, Spain

## Abstract

Seronegative antiphospholipid syndrome (SNAPS) is an autoimmune disease present in patients with clinical manifestations highly suggestive of Antiphospholipid Syndrome (APS) but with persistently negative consensus antiphospholipid antibodies (a-PL). IgA anti-**β**2 Glycoprotein I (aB2-GPI) antibodies are associated with APS. However, they are not currently considered to be laboratory criteria due to the heterogeneity of published works and the use of poor standardized diagnostic systems. We have aimed to assess aPL antibodies in a group of patients with clinical manifestations of APS (C-APS) to evaluate the importance of the presence of IgA aB2GPI antibodies in APS and its relation with other aPL antibodies. Only 14% of patients with C-APS were positive for any consensus antibody, whereas the presence of isolated IgA aB2GPI antibodies was found in 22% of C-APS patients. In patients with arterial thrombosis IgA aB2GPI, antibodies were the only aPL antibodies present. Serologic profile in primary APS (PAPS) is different from systemic autoimmune disorders associated APS (SAD-APS). IgA aB2GPI antibodies are more prevalent in PAPS and IgG aB2GPI antibodies are predominant in SAD-APS. The analysis of IgA aB2GPI antibodies in patients with clinical manifestations of PAPS might avoid underdiagnosed patients and provide a better diagnosis in patients with SAD-APS. Laboratory consensus criteria might consider including analysis of IgA aB2GPI for APS diagnosis.

## 1. Introduction

Antiphospholipid antibodies (aPL) are a heterogeneous group of autoantibodies directed against phospholipids, phospholipids complexed with proteins, or phospholipids binding proteins, localized on the membranes of endothelial cells, platelets, and other cells involved in the coagulation cascade [1, 2]. Antiphospholipid syndrome (APS) is an autoimmune multisystemic disorder characterized by recurrent thrombosis and pregnancy morbidity in patients with aPL antibodies [3]. APS was defined in the context of systemic autoimmune diseases as Systemic Lupus Erythematosus (SLE). However, shortly after, several authors suggested a separate category to group patients with APS clinical criteria and without systemic autoimmune disorders: the primary antiphospholipid syndrome (PAPS) [4, 5], currently the most common form of disease [6]. Patients with APS associated with systemic autoimmune disorders (also known as secondary antiphospholipid syndrome) were classified as SAD-APS [7]. Clinical criteria to diagnose APS include one or more episodes of arterial, venous, or small vessel thrombosis in any tissue or organ that must be confirmed by objective validated criteria as imaging studies or histopathology. Consensus APS pregnancy morbidity can be (1) unexplained death of a morphologically normal fetus at or beyond the 10th week of gestation, (2) premature births of a morphologically normal neonate before the 34th week of gestation because of eclampsia or severe preeclampsia or placental insufficiency, and (3) three or more unexplained consecutive spontaneous abortions before the 10th week of gestation. Laboratory criteria are (1) presence of Lupus anticoagulant (LA) in serum or plasma, (2) presence of anticardiolipin (aCL) antibodies IgG and/or IgM isotype in serum or plasma, and (3) presence of anti-*β*_2_ glycoprotein-I (aB2GPI) antibodies IgG and/or IgM isotype in serum or plasma. Antibodies should be present on two or more occasions at least 12 weeks apart. At least one clinical criterion and one laboratory criterion are needed for APS diagnosis [8]. Establishment of consensus criteria for APS allowed clinicians to standardize patient groups but also generated controversy. Several manifestations associated with antibodies aPL as heart valve disease, livedo reticularis (LR), aPL nephropathy, neurological manifestations, stroke, myocardial infarction, and thrombocytopenia were not included in the updated criteria [9, 10]. In addition, there are patients with clinical manifestations highly suggestive of APS but persistently negative for consensus aPL antibodies. These patients are classified as seronegative APS (snAPS) [11] and show similar clinical profile as seropositive patients [12].

In snAPS patients, recent works have revealed presence of aPL antibodies not included in APS criteria which might be relevant for the diagnosis of APS [13]. On the other hand, published aPL prevalence in the general population is highly heterogeneous, ranking between 1% and 5.6% in healthy subjects. Given these considerations, some authors have claimed that the current diagnostic criteria are too restrictive and of limited use for clinical purposes [14] and have suggested redefining APS [15].

Over the past few years much attention has been focused on the diagnostic value of IgA isotype aPL antibodies. Isolated IgA aB2GPI antibodies have been associated with APS on SLE patients [16] and with nonconsensus APS vascular pathology [17–19].

Although the majority of the published works have highlighted the value of IgA aB2GPI antibodies in APS diagnosis, there is controversy in the literature about the meaning of the presence of aB2GPI IgA antibodies. Insufficient standardization might be one of the causes and diagnostic tools are not sufficiently standardized. In addition, some works have been done with diagnostic assays that have not been optimized [20] that claim that IgA aB2GPI antibodies lack specificity in APS diagnosis and that do not provide useful clinical information. However, IgA aB2GPI antibodies have gained clinical relevance and were recently included as a clinic classification criterion for systemic lupus erythematosus [17]. Likewise, determination of IgA aB2GPI antibodies is recommended in patients with snAPS [21], LES, and in ethnics groups with a high prevalence of IgA isotype antibodies such as African Americans and long lasting SLE patients [18, 22].

In this work, we have studied a group of patients with clinical manifestations of APS (C-APS) to determine the presence of aPL consensus isotypes (IgG and IgM) and also the IgA isotype in order to evaluate diagnostic utility of IgA isotype antibodies detection.

## 2. Patients and Methods

### 2.1. Study Design

This work is a cross-sectional study carried out to assess the prevalence of APS and snAPS conditions and their association with aPL antibodies of IgG, IgM, and IgA isotypes.

The study complies with Spanish legislation and European Community directives for cross-sectional studies.

### 2.2. Patients

A total of 156 patients fulfilling clinical criteria for APS (independently of serological markers) were recruited out of the 902 patients referred by their physicians to the Immunology Department in the 12 de Octubre Hospital during a 5-month period (ending on November 4, 2013). Presence of serum aPL antibodies was analyzed. Clinical criteria for patient inclusion (Table 1) were venous thrombosis (VT), arterial thrombosis (AT), and pregnancy morbidity (PM). Pulmonary thromboembolisms were classified as VT. Patients with incomplete symptoms of APS (livedo reticularis, thrombocytopenia, abortions outside deadlines, etc.) or prothrombotic conditions secondary to other factors such as sepsis, homocystinemia, and genetic defects of coagulation factors (thrombin mutations, factor V Leiden, antithrombin deficiency, etc.) were ruled out. Women with gestational morbidity were studied to evaluate the cause of this problem. Chromosomal, anatomical, endocrine, infectious, immune, and thrombophilic factors were studied. All those women who presented any of the above factors with the exception of aPL were excluded from the study.

Mean age of the patients was 52.3 ± 1.8 years. The proportion of women was approximately 2 : 1 (65.4%: 102 women, 54 men). Fifteen patients (9.4%) had associated systemic autoimmune diseases: SLE: 13 (8.3%), systemic sclerosis (SS): 1, and rheumatoid arthritis + SLE: 1. These 15 patients were considered as SAD-APS. The remaining 141 had no association with any systemic autoimmune disease and were considered PAPS. Additional risk factors found in the patients were diabetes mellitus 16 patients (10.3%), hyperlipidemia 24 patients (15.4%), hypertension 48 patients (30.8%), Chronic kidney disease 3 patients (1.9%), surgery within the previous two months 3 patients (1.9%), and prolonged immobility 1 patient (0.6%).

Ethnicity of patients and controls was Mediterranean Caucasian in more than 97%.

### 2.3. Controls

The control group was randomly selected to represent the general population in our area. We included 306 sera from healthy blood donors, only considering one sample per patient because in our experience, a single determination of IgA aB2GPI antibodies has high diagnostic value [19]. In our laboratory, 3452 patients with at least 2 IgA aB2GPI determinations in the last 5 years were evaluated, 95.9% of whom had reproducible results.

Data of the patients and control were collected in an anonymized database. Sera samples were destroyed once the analysis was performed.

### 2.4. Laboratory Determinations

IgG/IgM aCL and aBGPI antibodies were measured using the BioPLex 2200 multiplex immunoassay system (Biorad, Hercules CA, USA). Antibody levels higher than 18 U/mL were considered positive following the manufacturer's guidelines.

IgA aCL and aBGPI antibodies were quantified by enzyme-linked immunosorbent assays (ELISA) using IgA-aCL and IgA-aB2GPI QUANTA Lite (INOVA Diagnostics Inc., San Diego, CA, USA). Antibody levels higher than 20 U/mL were considered positive following the manufacturer's guidelines.

Lupus anticoagulant (LA) activity was detected by coagulation assays, following the guidelines of the International Society on Thrombosis and Hemostasis (ISTH) [23]. We used the HemosIL dRVVT Screen, HemosIL dRVVT Confirm and HemosIL Silica Clotting Time assays (Instrumentation Laboratory SpA, Milano, Italy).

All serum samples were tested for IgG, IgM and IgA aCL and aB2GPI antibodies.

LA was determined in the 82 patients, which had been requested by their physicians (independently of APTT results). In addition, it was also determined in 8 additional patients who had APTT prolongation, although their physician had not requested it.

### 2.5. Statistical Methods

Results were expressed as mean ± standard error or absolute frequency and percentage. In scaled variables with two categories, comparisons were performed using the Student's *t*-test. Association between qualitative variables was determined with Pearson's Chi-square test or Fisher's exact test when appropriate. *P* values less than 0.05 were considered significant.

Data were processed and analyzed using the statistical program STATA 11 (StataCorp LP, College Station, TX, USA).

## 3. Results

### 3.1. aPL Antibody Levels and Their Relationship with C-APS Patients

**Figure 1:**
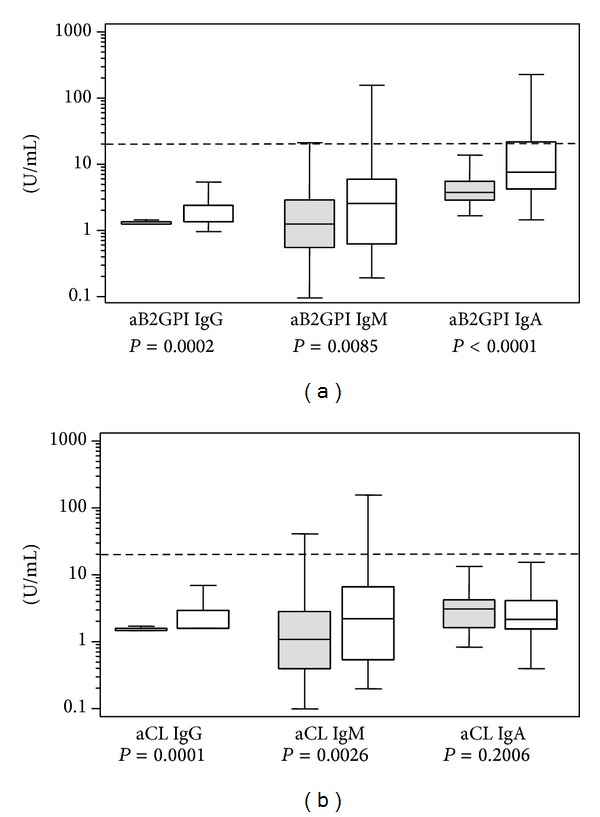
(a) Levels of anti-Beta 2 Glycoprotein I antibodies (aB2PGI) in controls (gray) and C-APS patients (white). (b) Levels of anticardiolipin antibodies (aCL) in controls (gray) and C-APS patients (white). Cutoff is indicated by a dotted line.

Mean levels of IgG, IgM, and IgA, both aCL and aB2GPI, antibodies were significantly higher in patients with C-APS than in controls (Table 2, Figures 1(a) and 1(b)).

Proportion of patients with aCL or aB2GPI antibodies of any isotype was significantly higher in C-APS patients than in controls (Table 3). IgA aB2GPI was the most prevalent antibody in C-APS patients (28.8%, Table 3). The main difference between C-APS patients and controls was found in IgA aB2GPI antibodies positivity, combined with any other aPL (odds ratio 24.4 *P* < 0.0001) or isolated (odds ratio 17.4 *P* < 0.0001, Table 3). On the C-APS group, only 22 patients (14.1%) were positive for any consensus aPL (IgG/IgM aCL or aB2GPI antibodies). Thirty-five patients (22.4%) were positive for isolated IgA aB2GPI antibodies and 45 patients (28.8%) were positive for IgA B2GPI antibodies combined with other isotypes (Table 3).

**Figure 2:**
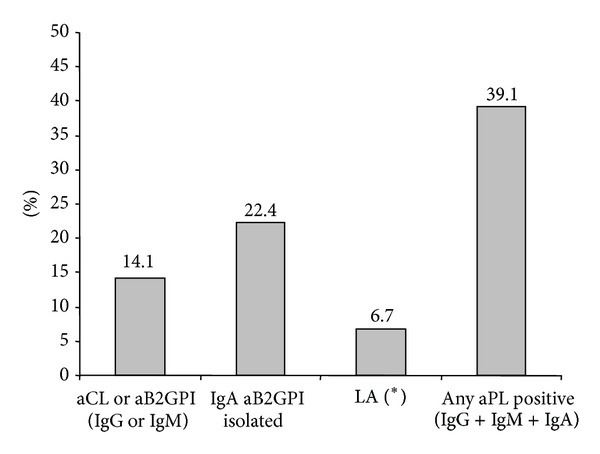
Percentage of C-APS patients positive for aPL antibodies. (*) LA detection was performed on 90 patients.

If we consider those with positivity of any aPL isotype antibodies (including IgA) as aPL positive patients, 61 patients would be positive (39.1%) due to the inclusion of 39 new patients who were positive for IgA isotype and negative for IgG and IgM (Figure 2).

Lupus anticoagulant was only positive for 6 of the 90 patients tested (6.7%, Figure 2). No significant associations with previously described risk factors were observed (not shown).

### 3.2. Prevalence of aPL Antibodies in PAPS and SAD-APS

SAD-APS patients were younger than PAPS ones (44.3 ± 3.0 versus 56.2 ± 1.7 years, *P* = 0.0021), with a greater percentage of women (93.3% versus 62.4%, *P* = 0.0200).

**Figure 3:**
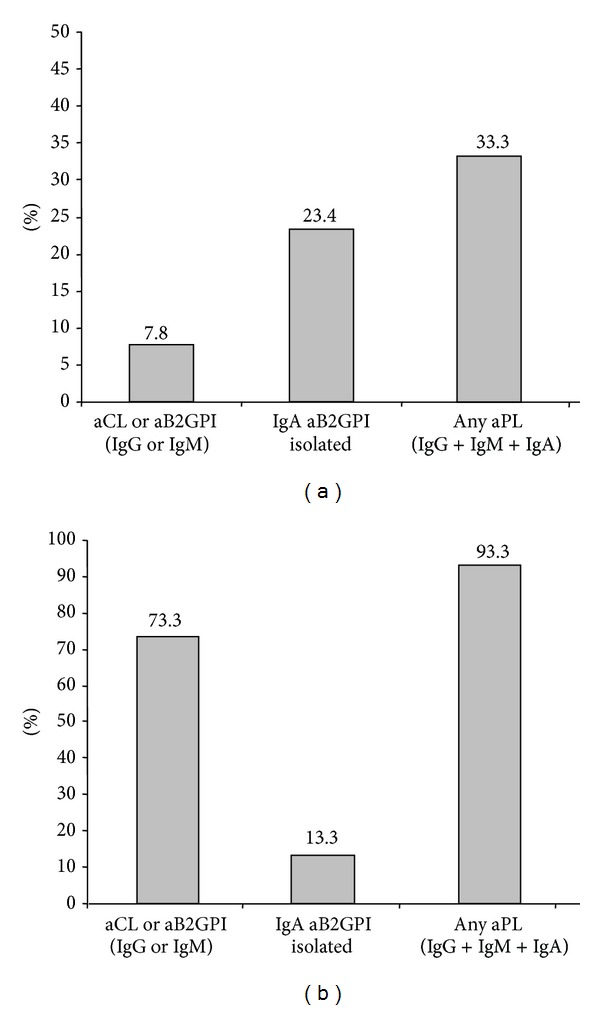
(a) Percentage of PAPS patients positive for aPL antibodies. (b) Percentage of SAD-APS patients positive for aPL antibodies.

Positivity of consensus aPL antibodies in SAD-APS patients was significantly higher than in patients with PAPS (Table 4, Figures 3(a) and 3(b)), especially for IgG isotype antibodies with odds ratios higher than 60 (*P* < 0.0001, Table 4). Positivity of IgA aB2GPI antibodies combined with other consensus aPL antibodies was also higher in SAD-APS patients (*P* = 0.0124) but isolated positivity of IgA aB2GPI antibodies did not show significant differences with PAPS group (*P* = 0.5732, Table 4).

Eleven (7.8%) PAPS patients were positive for consensus aPL antibodies isotypes. When IgA isotype positivity was also considered, 33.3% of the patients were seropositive (Figure 3(a)).

The most prevalent antibodies on PAPS patients were IgA aB2PGI (Table 4). Isolated IgA aB2GPI were the only positive antibodies in 70% of these seropositive patients.

Eleven (73.3%) of SAD-APS patients were positive for consensus isotypes aPL antibodies. When IgA isotype were included, 93.3% of patients were identified as seropositive. This improves the diagnostic capacity of consensus aPL but more discretely than in PAPS patients (Figure 3(b)). The most prevalent antibodies in SAD-APS patients were IgG aB2PGI (Table 4). No significant differences were observed between PAPS and SAD-APS patients regarding the clinical classification inclusion criteria (not shown).

### 3.3. Relationship between Clinical Manifestations of APS and aPL Antibodies

**Figure 4:**
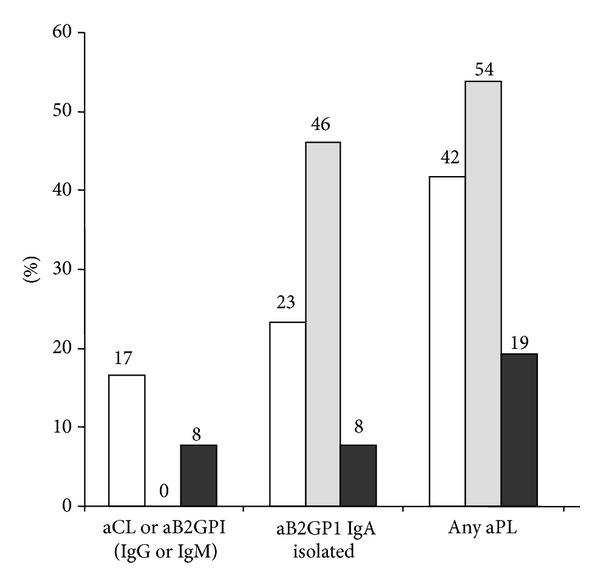
Percentage of APS patients positive for aPL antibodies. APS patients were classified as follows: venous thrombosis (white), arterial thrombosis (grey), and pregnancy morbidity (dark).

No differences between APS subgroups (VT, AT, and PM) were observed on aPL antibodies positivity (not shown). However, in patients with AT, IgA isotype antibodies were especially significant: 54% of the patients with AT were positive for IgA (odds ratio 70.2, *P* < 0.0001) and all patients with AT were negative for aPL antibodies of IgG and IgM isotypes (Table 5, Figure 4).

## 4. Discussion

Assessment of IgA isotype aPL antibodies, especially anti B2GPI, allowed clinicians to identify more patients with C-APS as seropositive [24], detecting up to nearly 40% of the cases while using Sapporo's consensus criteria of laboratory diagnosis only detected 14.1% of the cases.

The prevalence of aPL autoantibodies in the control group was similar to previously reported studies for IgG and IgM isotype [9, 25] and also for IgA isotype [26]. Most patients positive for IgA anti B2GPI antibodies were negative for IgA aCL. The proportion of IgA aB2GPI positive versus IgA aCL positive was also similar to that previously published [16].

It stands out that only 9.4% of our patients with APS symptoms had SAD-APS when could be expected close to 50% according to the published data [6]. A possible explanation for this difference is because we were studying patients with C-APS and the published studies have only evaluated seropositive APS patients. If we limit our study only to the 22 patients positive for consensus aPL antibodies, patients with SAD-APS would be 50% (11), this being in accordance with the expected prevalence. This observation emphasizes that the laboratory criteria for APS were designed to achieve greater specificity in the SAD-APS, resulting in the disadvantage that cases of PAPS remain underdiagnosed [27].

The aPL antibodies profile differs for PAPS patients than for SAD-APS patients. Whereas in SAD-APS patients the most prevalent antibody is IgG isotype (aB2GPI and aCL), it is the IgA isotype in PAPS patients. Diagnostic utility of isolated IgA aB2GPI antibodies in patients with C-APS was previously reported in a small cohort of patients [22]; Our study has been carried out with a larger number of patients without any selection bias.

Incorporating the IgA isotype into the diagnostic guidelines could be especially useful in patients with PAPS. It would make it possible to identify up to 4 times more patients who are not considered as APS with the current diagnosis criteria. This change in criterion might be a diagnostic improvement, especially for patients with AT [28] who are negative for consensus aPL antibodies in our study.

If IgA isotype antibodies are taken into consideration as consensus aPL antibodies, about 27% more patients would be identified as SAD-APS. Although IgA isotype is less relevant in SAD-APS than PAPS, it has greater utility than that provided by the IgM isotype, as was observed previously [18].

The relevance of IgA aB2GPI antibodies in patients with SLE was recently accepted as an inclusion diagnostic criterion for SLE by the Systemic Lupus International Collaborating Clinics Classification Criteria for Systemic Lupus Erythematosus [17]. It is unknown how the immune response of IgA antibodies against B2GPI is generated. It has been hypothesized that anti-B2GPI response could occur by molecular mimicry between pathogens and B2GPI epitopes [3, 29] or within the context of an autoimmune syndrome induced by adjuvants (ASIA) [30]. If infection or antigen presentation takes place in the respiratory or digestive tracts, the mucosal immune system directs the response of the antibodies towards the production of the IgA isotype.

The mechanisms by which IgA aB2GPI antibodies can cause thrombosis remain unknown. Several pathogenic pathways have been suggested as hypercoagulable state secondary to activation of the complement system [31], inhibition of the fibrinolytic system [32], and cellular activation of platelets, monocytes, and endothelial cells (EC) [27, 33]. B2GPI is localized on the level of the cell surface of human EC associated with lipids or membrane proteins [34]. aB2GPI antibodies can activate EC [35], upregulate adhesion molecules, and induce cytokine production [36]. Cell activation by aPL antibodies appears to be a major pathogenic cause in the pathogenesis of APS [37]. As human IgA cannot fix the complement using the classical pathway [38], in the case of patients with isolated positivity of IgA aB2GPI this mechanism takes on special relevance.

If we consider the presence of IgA B2GPI antibodies as laboratory diagnostic criteria together with IgG and IgM antibodies, we can increase the number of APS patients diagnosed. However, 61% of C-APS patients could not be identified as seropositive.

In the near future, the determination of other less prevalent APL antibodies, as antiAnnexin V and antiphosphatidylserine/Prothrombin should be evaluated in order to identify more APS patients who are currently misdiagnosed.

Antibodies aB2GPI of IgA isotype are present in 1–3% of the healthy population. Even though these antibodies could be considered as an epiphenomenon, their importance has not been established yet. Prospective studies are needed to clarify their predictive value in vascular and thromboembolic events.

Most studies of aPL are made on series of patients with systemic autoimmune diseases (SAD-APS) and tend to extrapolate their conclusions to all patients with APS.

In this study we have focused on patients with clearly defined C-APS independently of the presence or not of underlying systemic autoimmune diseases. Therefore, we have been able to obtain a better idea of the importance of the C-APS in the clinical practice.

These results should be confirmed in multicenter studies that make it possible to manage larger groups of patients and would help to assess other APS-associated manifestations.

Our studies suggest that the serological profile of patients with PAPS (IgA is the most prevalent isotype) is different from the SAD-APS (IgG is the most prevalent isotype). The assessment of IgA aB2GPI antibodies in patients with suspected PAPS is important to identify, treat, and manage patients who, in accordance with the current criteria, are not diagnosed at this point in time of the disease. This is not as important as PAPS in the case of SAD-APS because patients are monitored regularly in the context of their underlying disease and any clinical event is quickly detected.

We agree with other authors that the classification criteria for APS should be revised to include IgA aB2GPI antibodies in patients with SLE [18] but perhaps it is even more important to include these criteria in PAPS.

## Figures and Tables

**Table 1:** Clinical criteria of inclusion in APS patients group and prevalence.

Clinical criteria	Patients
Venous thrombosis	117 (75.0%)
Arterial thrombosis	11 (7.0%)
Venous and arterial thrombosis	2 (1.3%)
Pregnancy morbidity	25 (16.0%)
Venous thrombosis and pregnancy morbidity	1 (0.6%)

**Table 2:** Quantitative values (U/mL) of autoantibodies aPL in patients with symptoms of APS versus control population.

Antibody	Control		APS		
	Mean	S.E.	Mean	S.E.	*P* value
aB2GPI IgG	1.6	0.07	14.6	3.35	0.0002
aB2GPI IgM	2.6	1.87	7.6	0.30	0.0085
aB2GPI IgA	5.5	0.52	26.9	3.94	<0.0001
aCL IgG	2.0	3.28	14.8	0.10	0.0001
aCL IgM	2.2	0.20	6.4	1.34	0.0026
aCL IgA	3.5	0.95	8.2	3.20	0.2006

**Table 3:** Positive aPL antibodies in C-APS patients versus controls.

	Controls (*n* = 306)	APS (*n* = 156)	OR	*P*
aB2GPI IgG	2 (0.6%)	14 (8.9%)	14.9	<0.0001
aB2GPI IgM	3 (0.9%)	8 (5.1%)	5.4	0.0146
aB2GPI IgA	5 (1.6%)	45 (28.8%)	24.4	<0.0001
aCL IgG	3 (0.9%)	17 (10.8%)	12.3	<0.0001
aCL IgM	2 (0.6%)	8 (5.1%)	8.2	0.0053
aCL IgA	3 (0.9%)	8 (5.1%)	5.5	0.0148
aB2GP1 IgA (isolated)	5 (1.6%)	35 (22.4%)	17.4	<0.0001
aCL or aB2GPI (IgG or IgM)	6 (2.0%)	22 (14.1%)	8.2	<0.0001
aCL or aB2GPI any isotype	13 (4.2%)	61 (39.1%)	14.5	<0.0001

**Table 4:** Positive aPL antibodies in PAPS versus SAD-APS patients.

Antibody	PAPS (*n* = 141)	SAD-APS (*n* = 15)	OR SAD-APS	*P*
aB2GPI IgG	3 (2.1%)	11 (73.3%)	126.5	<0.0001
aB2GPI IgM	5 (3.5%)	3 (20%)	6.8	0.0331
aB2GPI IgA	36 (21.9%)	9 (60%)	4.4	0.0124
aCL IgG	6 (4.2%)	11 (73.3%)	61.9	<0.0001
aCL IgM	5 (3.5%)	3 (20%)	6.8	0.0331
aCL IgA	4 (2.8%)	4 (26.7%)	12.4	0.0008
aB2GP1 IgA (isolated)	33 (23.4%)	2 (13.3%)	0.5	0.5732
aCL or aB2GPI (IgG or IgM)	11 (7.8%)	11 (73.3%)	32.5	<0.0001
aCL or aB2GPI any isotype	47 (33.3%)	14 (93.3%)	28.0	<0.0001

**Table 5:** APS morbidity and aPL autoantibodies.

Antibodies	Venous thrombosis			Arterial thrombosis			Pregnancy morbidity		
	*N*	OR	*P*	*N*	OR	*P*	*N*	OR	*P*
aB2GPI IgG	12 (10%)	16.9	<0.0001	0 (0%)	0	0.1332	2 (8%)	12.6	0.0262
aB2GPI IgM	7 (6%)	6.2	0.0087	0 (0%)	0	0.2677	1 (4%)	4	0.7265
aB2GPI IgA	36 (30%)	25.8	<0.0001	7 (54%)	70.2	<0.0001	3 (12%)	7.8	0.0125
aCL IgG	15 (13%)	14.4	<0.0001	0 (0%)	0	0.2677	2 (4%)	8.4	0.0630
aCL IgM	7 (6%)	9.4	0.0029	0 (0%)	0	0.1332	1 (8%)	6	0.5671
aCL IgA	7 (6%)	6.2	0.0087	1 (0%)	0	0.2677	1 (4%)	4	0.7265
aB2GP1 IgA (isolated)	28 (23%)	18.3	<0.0001	6 (46%)	51.6	<0.0001	2 (8%)	5	0.1759
aCL or aB2GPI (IgG or IgM)	20 (17%)	10	<0.0001	0 (0%)	0	0.4994	2 (8%)	4.1	0.2445
aCL or aB2GPI any isotype	50 (42%)	16.1	<0.0001	7 (54%)	26.2	<0.0001	5 (19%)	5.3	0.0053
